# Tetanus in Civilian Practice

**Published:** 1918-02-02

**Authors:** 


### TETANUS IN CIVILIAN PRACTICE.

Under this heading a recent number of the *Lancet* drew attention to the occurrence of tetanus among members of the civilian community, and to the need for the employment of a prophylactic serum in every instance where there is a chance that a wound may be followed by this terrible complication. The deadly character of tetanus received a widespread advertisement in the early days of the war, and people generally became aware that it is due to a germ which is present in the soil, and that consequently wounds which become contaminated by contact with earth or mud give an opportunity for the entrance of this germ into the body and for the development of tetanus. Whether the wound is slight or severe is not of much moment; the risk arises from the contamination just described. The war obviously afforded many opportunities for this risk, and hence many cases of tetanus occurred, with a corresponding loss of life. To prevent such a disaster was a matter of the greatest importance, and fortunately preventive medicine proved itself equal to the event. That there was some hope in the curative treatment of these cases by the use of an appropriate serum was a general belief, but for the most part this line of attack had not proved satisfactory. Only too often, when the symptoms of the disease once appeared, all methods of treatment proved ineffective. The suggestion therefore arose that a serum should be used as a prophylactic, which meant in practice that it should be injected into every patient whose wounds were at all likely to have received an infection by the germ of tetanus. It is well known that between the infliction of the wound and the appearance of symptoms of tetanus an interval, perhaps of several days, elapses, and the argument was that this interval should be utilised by the injection of an antitetanic serum in the hope of preventing the development of the disease. This policy has been highly successful, as the Army records show, and the dread of tetanus as a wound complication in military hospitals has largely disappeared.

Why should the same practice not be adopted in civilian practice? This is what our contemporary proposes, and we cordially support the suggestion. Cases of tetanus, especially among gardeners and others who work on the soil, are more numerous than is generally known, and coroners are by no means unfamiliar with them. The records generally show that no serum has been used until muscular spasms were recognised. But this is too late for efficiency. The only alternative is to use the serum promptly on the recognition of any wound where the risk of tetanus is involved. Doubtless in many cases the procedure would be unnecessary. But there is no means of distinguishing in advance the infected from the uninfected cases, and hence safety can be secured only by treating them all. This procedure would involve some expense, but the deaths from tetanus in civil life, judging from the Army results, would largely disappear.

